# Asymmetric auxin distribution establishes a contrasting pattern of aerenchyma formation in the nodal roots of *Zea nicaraguensis* during gravistimulation

**DOI:** 10.3389/fpls.2023.1133009

**Published:** 2023-04-19

**Authors:** Jiayang Ning, Takaki Yamauchi, Hirokazu Takahashi, Fumie Omori, Yoshiro Mano, Mikio Nakazono

**Affiliations:** ^1^Graduate School of Bioagricultural Sciences, Nagoya University, Nagoya, Aichi, Japan; ^2^Bioscience and Biotechnology Center, Nagoya University, Nagoya, Aichi, Japan; ^3^Division of Feed and Livestock Research, National Agriculture and Food Research Organization (NARO) Institute of Livestock and Grassland Science, Nasushiobara, Tochigi, Japan; ^4^The University of Western Australia (UWA) School of Agriculture and Environment, The University of Western Australia, Crawley, WA, Australia

**Keywords:** aerenchyma, auxin distribution, cell death, gravistimulation, lateral root, laser microdissection, maize (*Zea mays* ssp. *mays*), Z*ea nicaraguensis*

## Abstract

Auxin distribution is essential for determining root developmental patterns. The formation of lateral roots and constitutive aerenchyma, which is a gas space developed through cell death, is regulated by auxin in rice (*Oryza sativa*). However, it is unclear whether the involvement of auxin in constitutive aerenchyma formation is conserved in other species. In this study, we found that constitutive aerenchyma formation was regulated by auxin in the nodal roots of *Zea nicaraguensis*, a wild relative of maize (*Zea mays* ssp. *mays*) grown naturally on frequently flooded coastal plains. Subsequent gravistimulation (root rotation) experiments showed opposite patterns of aerenchyma and lateral root formation. Lateral root formation on the convex side of rotated roots is known to be stimulated by a transient increase in auxin level in the pericycle. We found that aerenchyma formation was accelerated in the cortex on the concave side of the rotated nodal roots of *Z*. *nicaraguensis*. A cortex-specific expression analysis of auxin-responsive genes suggested that the auxin level was higher on the concave side than on the convex side. These results suggest that asymmetric auxin distribution underlies the regulation of aerenchyma and lateral root formation in the nodal roots of *Z*. *nicaraguensis*. As aerenchyma reduces the respiratory cost of the roots, constitutive aerenchyma on the concave side of the nodal root may balance resource allocation, thereby contributing to the uptake of water and nutrients by newly formed lateral roots. Our study provides insights into auxin-dependent asymmetric root patterning such as that of gravistimulation and hydropatterning response.

## Introduction

Excess water from heavy rainfall causes soil flooding, which restricts gas diffusion into submerged soil (Colmer, 2003; Voesenek and Bailey-Serres, 2015). The roots of upland plants such as maize (*Zea mays* ssp. *mays*) and wheat (*Triticum aestivum*) suffer from oxygen deficiency during flooding (Colmer and Voesenek, 2009; Yamauchi et al., 2018; Lin et al., 2021; Pedersen et al., 2021). Under soil flooding, some plants form aerenchyma consisting of gas spaces that facilitate oxygen diffusion from the shoot to the submerged roots (Armstrong, 1979; Colmer, 2003). Lysigenous aerenchyma is formed through cell death and the subsequent lysis of cortical cells (Yamauchi and Nakazono, 2022a). In rice (*Oryza sativa*), lysigenous aerenchyma is constitutively formed under aerobic conditions and further induced in response to oxygen deficiency (Colmer et al., 2006; Shiono et al., 2011). The former is referred to as constitutive aerenchyma (CA) formation and the latter as inducible aerenchyma formation (Colmer and Voesenek, 2009). By contrast, the formation of aerenchyma in the roots of upland plants such as maize and wheat is low under aerobic conditions, but it is induced in response to oxygen deficiency (Gong et al., 2019; Yamauchi et al., 2019a).

*Z. nicaraguensis* is a wild relative of maize found in flooded lowlands on the coastal plain of Nicaragua (Iltis and Benz, 2000). *Z. nicaraguensis* can be crossed with maize as a useful genetic resource for improving flooding tolerance in maize (Mano et al., 2009; Mano et al., 2016; Gong et al., 2019). *Z. nicaraguensis* forms CA and a barrier to radial oxygen loss, both of which confer flooding tolerance (Abiko et al., 2012; Mano and Nakazono, 2021). Quantitative trait loci (QTLs) associated with CA formation in *Z. nicaraguensis* have been identified using populations of maize inbred lines Mi29 and B64 crossed with *Z. nicaraguensis* (Mano et al., 2007; Mano and Omori, 2008; Mano et al., 2009). Gong et al. (2019) demonstrated high flooding tolerance of the pyramided line IL-AE91, which possesses four QTLs for CA formation, compared to Mi29 under oxygen-deficient conditions or flooded soil.

During drought and nutrient deficiency conditions, aerenchyma formation reduces the respiratory cost per volume of roots (Lynch, 2018; Lynch, 2019; Schneider et al., 2020a; Schneider et al., 2020b). Root cortical senescence in barley (*Hordeum vulgare*) reduces the nutrient requirement of roots for optimal plant growth under suboptimal availability of nitrogen, phosphorus, and potassium owing to a reduction in root respiration and an increase in total carbon reserves (Schneider et al., 2017a; Schneider et al., 2017b). A simulation study predicted that aerenchyma contributes to the growth of maize and common bean (*Phaseolus vulgaris*) under suboptimal phosphorus availability (Postma and Lynch, 2011b). Recombinant inbred lines of maize with increased aerenchyma formation show deeper rooting and higher yields than those with reduced aerenchyma formation under drought conditions (Zhu et al., 2010). This finding suggests that aerenchyma formation contributes to the efficient allocation of energy resources. Indeed, aerenchyma formation correlates with root length density in deep soil layers (Zhu et al., 2010). Another simulation study predicted that aerenchyma is beneficial for the growth of maize genotypes with greater lateral branching density, even though the effect of aerenchyma depends on the type and degree of nutrient deficiency (Postma and Lynch, 2011a).

As auxin is a common regulator of aerenchyma and lateral root (LR) formation (Yamauchi et al., 2019b), a close physiological relationship may exist between them. Root gravitropism, primarily controlled through the expression and localization of auxin efflux carrier PIN-FORMED (PIN) family proteins, is essential for determining root growth angles in plants (Morita and Tasaka, 2004; Petrasek and Friml, 2009; Tanaka et al., 2022). In the gravity-stimulated (rotated) primary roots of *Arabidopsis*, PIN3 and PIN7 localize to the lower (concave) side of the columella cells to transport auxin in the direction of gravity (Han et al., 2021). PIN2 expression is higher on the concave side and stimulates auxin transport from the columella to the elongation zone (Han et al., 2021). Moreover, a rice mutant defective in PIN2 function displayed curly root phenotypes caused by reduced auxin flow back to the elongation zone (Inahashi et al., 2018). A higher auxin level on the concave side of rotated roots is required for the suppression of cell elongation for downward growth (Laskowski et al., 2008), whereas an increase in auxin level overtime on the upper (convex) side stimulates the initiation of LR primordia in rotated roots (Richter et al., 2009; Lucob-Agustin et al., 2020). By contrast, the regulation of CA formation during gravistimulation remains unclear.

It is unclear whether the involvement of auxin in CA formation is conserved in wetland plant species. To test this hypothesis, we examined whether treatment with the natural auxin indole-3-acetic acid (IAA) and/or the polar auxin transport inhibitor *N*-1-naphthylphthalamic acid (NPA) affects CA formation in the nodal roots of *Z. nicaraguensis*. Modeling-based age-dependent analysis was performed to reveal the accurate patterns of aerenchyma formation. Based on the assumption that a longitudinal auxin gradient is required for initiating CA formation, we evaluated the transverse pattern of CA formation in the gravity-stimulated nodal roots. Moreover, the auxin distribution pattern was predicted through an analysis of the expression of auxin-responsive genes in the root cortex.

## Materials and methods

### Plant materials and growth conditions

Seeds of *Z. nicaraguensis* were treated with 10% (v/v) hydrogen peroxide solution for 2 min to sterilize and align with the time of germination (Naredo et al., 1998). The seeds were then placed on 5-cm-diameter filter paper (3MM CHR; Whatman) in a Petri dish soaked with deionized water in a growth chamber (LJ-411SP; Nippon Medical & Chemical Instruments Co., Ltd) at 28°C under dark conditions. After 2 days, the germinated seedlings were placed 5 cm from the upper end of 25 × 25 cm^2^ filter papers. Subsequently, the filter papers including the seedlings were rolled and transferred to 2-L gray plastic pots (250 mm × 120 mm × 90 mm) containing 200 mL of deionized water. The pots were then covered with aluminum foil and placed in a growth chamber (LJ-411SP; Nippon Medical & Chemical Instruments Co., Ltd) for 4 days at 28°C under dark conditions. After 4 days, the aluminum foil was removed and the shoots were exposed to light for 1 day [relative humidity of 50%–60%, photosynthetically active radiation (PAR) of 250–300 μmol m^−2^ s^−1^, 28°C for 14 h under light and 25°C for 10 h in dark]. Five-day-old seedlings were fixed with a soft gray sponge and transferred to 5-L gray plastic pots (240 mm × 180 mm × 120 mm; four plants per pot) containing half-strength hydroponic nutrient solution [28°C under light (PAR, 200–250 µmol m^−2^ s^−1^) for 14 h and 25°C in dark for 8 h]. After 7 days, the seedlings were transferred to new 5-L gray plastic pots containing full-strength nutrient solution [28°C under light (PAR, 200–250 µmol m^−2^ s^−1^) for 14 h and 25°C in dark for 8 h]. The full-strength nutrient solution comprised 1.0 mM NH_4_NO_3_, 0.5 mM NaH_2_PO_4_, 0.3 mM K_2_SO_4_, 0.3 mM CaCl_2_, 0.6 mM MgCl_2_, 0.045 mM Fe-EDTA, 50 μM H_3_BO_3_, 9 μM MnSO_4_, 0.3 μM CuSO_4_, 0.7 μM ZnSO_4_, and 0.1 μM Na_2_MoO_4_ (Mae and Ohira, 1981). In addition, 5.0 mM 2-(N-morpholino) ethanesulfonic acid (MES) was added, and the pH of the solution was adjusted to 5.5 using an appropriate amount of 10 N KOH (Abiko et al., 2012). The nutrient solution was continuously aerated to maintain a dissolved oxygen level higher than 7.0 mg L^−1^. To avoid iron deficiency, 1 mL of 25 mM FeSO_4_ was added every 2 days to maintain the Fe^2+^ concentration at 5.0 μM (Kulichikhin et al., 2014).

### Chemical treatments

For IAA and/or NPA treatment(s), 20-day-old aerobically grown *Z. nicaraguensis* seedlings were transferred to 5-L gray plastic pots containing full-strength nutrient solution with 5 µM IAA and/or 0.05 µM NPA and grown for an additional 48 h. Subsequently, 25 µL of 1 M IAA (Sigma-Aldrich) and/or 2.5 µL of 100 mM NPA (Sigma-Aldrich) were added to prepare 5 L of nutrient solution with 5 µM IAA and/or 0.05 µM NPA, respectively. As the stock solutions of IAA and NPA were dissolved in dimethylformamide (DMF), an equal amount of DMF was added to the nutrient solutions used for the control and treatments. The concentrations of IAA and NPA were determined based on the concentrations used in a previous study (Yamauchi et al., 2019b).

### Gravistimulation treatment

For the gravistimulation treatment, uncovered 1.5-L plastic transparent plates (300 mm × 220 mm × 25 mm) containing 700 mL of 1.0% (w/v) agar (Wako) were prepared. The single third nodal roots (of length 30–40 mm) were selected from the 20-day-old *Z. nicaraguensis* seedlings, and the remaining roots were removed by cutting with a pair of forceps. Subsequently, the seedlings with single nodal roots were fixed on agar plates. The root parts were covered with a wet paper towel and plastic wrap to prevent drying, and then covered with aluminum foil (**Supplementary Figure S1**). The nodal roots were kept perpendicular to the vertical plane for the gravistimulation treatment and parallel to the vertical plane for the control treatment (**Supplementary Figure S1**). Sampling was conducted 24, 48, and 72 h after the start of the gravistimulation treatments.

### 4’,6-Diamidino-2-Phenylindole staining

To detect and count LR primordia in the bent regions of the third nodal roots, 10-mm-long root segments were hydrolyzed twice with 4% paraformaldehyde fixing solution under vacuum for 30 min and then washed twice with a solution of 1× phosphate-buffered saline (PBS; Cold Spring Harbour Laboratories, 2006) under vacuum for 5 min at 25°C. Subsequently, the solution was replaced with 0.5% Triton and incubated for 20 min at room temperature, and then washed twice with 1× PBS. Thereafter, the root segments were incubated in 0.5% DAPI (Dojindo) for 20 min in the dark and washed twice with 1× PBS. Finally, the root segments were hyalinized with methyl salicylate, and the stained LR primordia were photographed under a microscope (LEICAM165FC) with a CCD camera (DP70; Olympus). DAPI was excited with a 405-nm laser.

### Anatomical observation

For the anatomical observation following the chemical treatments, cross-sections of 4-mm-long segments excised from the third nodal roots of *Z. nicaraguensis* were prepared. The root segments at 30, 40, 50, 60, 70, and 80 mm (± 2 mm) from the tips were cut from approximately 100-mm-long nodal roots. For the gravistimulation experiment, the nodal roots were marked by cutting a narrow groove at the top on the convex side of the roots using a razor blade to distinguish the concave and convex sides of the roots. After the treatment, cross-sections from the 4-mm-long segments excised from each position of the nodal roots were prepared. The root segments were prepared at 0 mm (bent part), −10 mm (direction toward the root base), and +10 mm (direction toward the root tip) (± 2 mm). The cross-sections were prepared by hand sectioning the roots using a razor blade and photographed using an optical microscope (BX60; Olympus) with a CCD camera (DP70; Olympus). The aerenchyma proportion in each cross-section was measured using ImageJ software (version 1.51j8; National Institutes of Health).

### Laser microdissection

Root segments of length 5 mm were obtained from the bent parts of the third nodal roots of *Z*. *nicaraguensis* and fixed in 100% methanol. After fixation, the samples were embedded in paraffin and sectioned to a thickness of 12 µm. Serial transverse sections were placed on polyethylene naphthalate (PEN) membrane glass slides (Thermo Fisher Scientific). The paraffin on the sections was removed by immersing the slides in 100% Histoclear II (National Diagnostics) for 10 min, followed by air-drying at room temperature. The sections were then subjected to laser microdissection (LM) as described by Takahashi et al. (2010). The cortex on the concave and convex sides of the rotated third nodal roots were collected from the tissue sections using a Zeiss PALM MicroBeam Laser Microdissection System (Carl Zeiss).

### RNA extraction

Total RNA was extracted from LM-isolated tissues using a PicoPure RNA isolation kit (Thermo Fisher Scientific) according to the manufacturer’s instructions. The extracted total RNA was quantified using a Quant-iT™ RiboGreen RNA reagent and kit (Invitrogen) according to the manufacturer’s instructions. The quality of the total RNA was evaluated using an RNA 6000 Pico kit and an Agilent 2100 Bioanalyzer (Agilent Technologies), as described by Takahashi et al. (2010). The RNA integrity number (RIN) of the samples is shown in **Supplementary Figure S2**.

### Quantitative reverse transcription-polymerase chain reaction

Total RNA (100 pg) was used as a template, and transcript levels were measured using a One Step SYBR Prime Script RT-PCR Kit II (Takara Bio Inc.) and StepOnePlus Real-Time PCR System (Applied Biosystems) according to the manufacturer’s instructions. The PCR fragments of each gene were purified, quantified, and used to generate standard curves for absolute quantification. The primer sequences are listed in **Supplementary Table S1**.

### Regression model analysis

Gompertz curve fitting was conducted using standard statistical tools in R software (version 3.5.2; https://rdrr.io/r/stats/stats-package.html). For model fitting, the percentage of aerenchyma formed was used as the response variable and the distance from the root tips was used as the explanatory variable, according to Yamauchi et al. (2020a). The percentage of aerenchyma formed at each time point was calculated using the equation obtained *via* Gompertz curve fitting using the distance from the root tip at each time point as the explanatory variable. The distance from the root tip at each time point was calculated using the corresponding elongation rates.

### Statistical analysis

Statistical differences between means were calculated using a two-sample *t*-test. Multiple comparisons were analyzed using a one-way ANOVA and the least significant difference (LSD) test with SPSS Statistics Version 19 (IBM Software, New York, USA). Statistical significance was set at *p* < 0.05.

## Results

### Effect of auxin and/or auxin transport inhibitor on aerenchyma formation in the nodal roots

**Figure 1:**
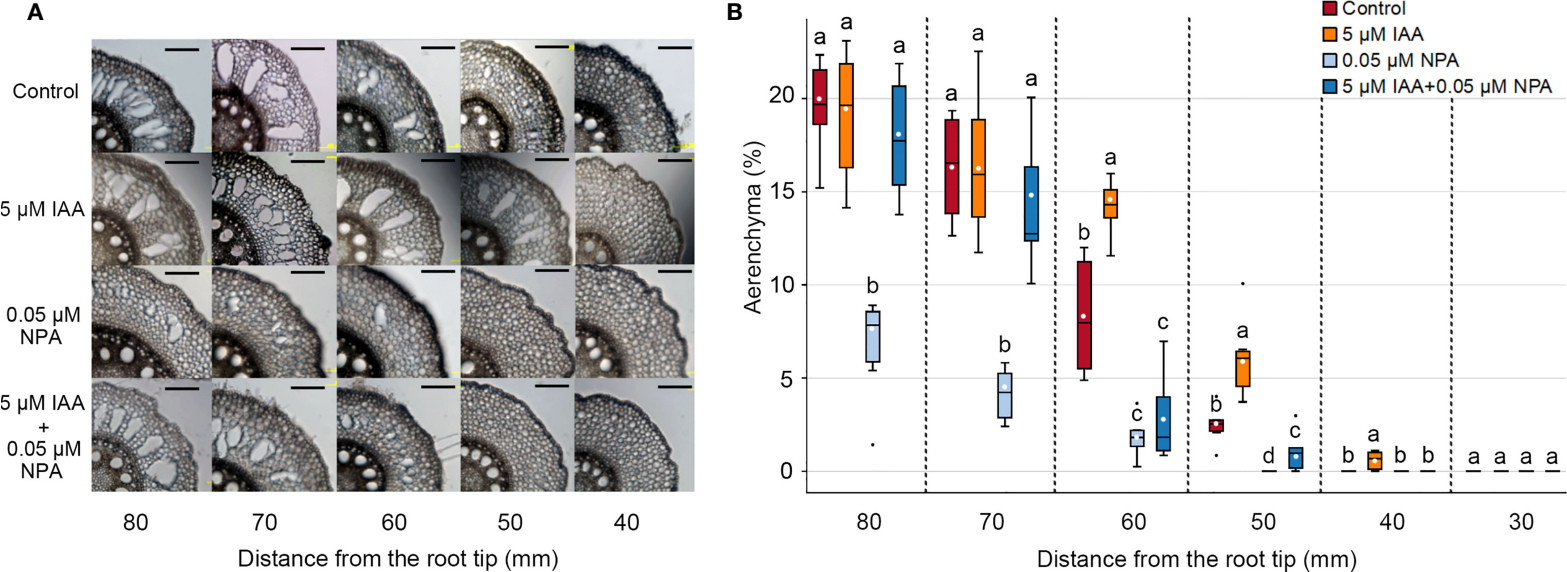
Aerenchyma formation in the third nodal roots of *Z. nicaraguensis* under aerated conditions with IAA and/or NPA treatments. **(A)** Cross-sections at 40, 50, 60, 70, and 80 mm from the tips of third nodal roots with or without 5 µM IAA and 0.05 µM NPA treatments. Scale bar = 200 μm. **(B)** Percentage of aerenchyma in root cross-sectional areas at 30, 40, 50, 60, 70, and 80 mm from the root tips. Boxplots show the median (horizontal lines), 25^th^ to 75^th^ percentiles (edge of the boxes), minimum to maximum (edge of the whiskers), and mean values (dots in the boxes) (*n* = 6). Significant differences among the treatments are denoted with different lowercase letters (*p* < 0.05, one-way ANOVA, followed by LSD test for multiple comparisons).

To understand the involvement of auxin in CA formation, we evaluated the effects of endogenous auxin (IAA) and/or a polar auxin transport inhibitor (NPA) on aerenchyma formation in the third nodal root of *Z. nicaraguensis*. Twenty-day-old aerobically grown seedlings of *Z. nicaraguensis* were treated with 5 μM IAA and/or 0.05 μM NPA for 2 days. In the control, aerenchyma formation was initiated 50 mm from the root tips and gradually increased toward the basal part of the nodal roots (**Figure 1A**). In the IAA treatment, aerenchyma formation was first detected at 40 mm (**Figure 1B**), and the percentage of aerenchyma formed at 50 and 60 mm was significantly higher than that in the control (**Figure 1B**). However, there were no significant differences in the percentage of aerenchyma formed between the IAA treatment and control at 70 and 80 mm from the root tips (**Figure 1B**). By contrast, the percentage of aerenchyma formed was remarkably lower in the NPA treatment than in the control at 50 to 80 mm from the root tips (**Figure 1B**). Interestingly, the IAA treatment restored this reduction in aerenchyma formation by the NPA treatment at 70 and 80 mm, but it did not completely restore the reduction at 50 and 60 mm (**Figure 1B**). This result could be attributed to the more pronounced effect of NPA on the more apical part of the nodal roots.

### Analysis of age-dependent aerenchyma formation in the nodal roots

**Figure 2:**
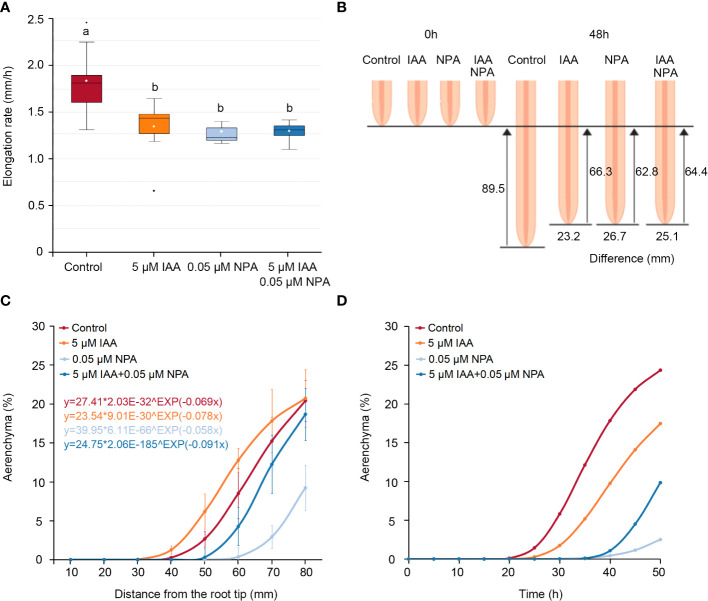
Age-dependent aerenchyma formation in the third nodal roots of *Z. nicaraguensis* under aerated conditions with IAA and/or NPA treatments. **(A)** Elongation rate of the nodal roots of *Z. nicaraguensis* seedlings grown under aerated conditions with IAA and/or NPA treatments. Boxplots show the median (horizontal lines), 25^th^ to 75^th^ percentiles (edge of the boxes), minimum to maximum (edge of the whiskers), and mean values (dots in the boxes) (*n* = 6). Significant differences among the treatments are denoted with different lowercase letters (*p* < 0.05, one-way ANOVA, followed by LSD test for multiple comparisons). **(B)** Schematic diagram of root elongation and differences in the distances from the root tips among the treatments at 0 h and 48 h. **(C)** The Gompertz curve is fitted for the percentage of aerenchyma formed. The obtained equations for each of the treatments are indicated above the graph. **(D)** Age-dependent aerenchyma formed was predicted by the equations obtained from Gompertz curve fittings. The percentage of aerenchyma formed at each time point was calculated using the distance from the root tips at each time point as the explanatory variable, which was calculated from the elongation rate under each treatment **(A)**.

At the same distance from the nodal root tips in *Z. nicaraguensis*, the IAA and/or NPA treatments affected the percentage of aerenchyma formed (**Figure 1**), but the NPA and IAA treatments significantly reduced the root elongation rate (**Figure 2A**). As a result, the root-elongation rate in the control in 2 days was 23.2, 26.7, and 25.1 mm higher than that in the IAA, NPA, and IAA and NPA treatments, respectively (**Figure 2B**). This result suggests that different elongation rates among the treatments affected the age of the root cells and masked the effects of IAA and NPA on aerenchyma formation. Recently, Yamauchi et al. (2020a) demonstrated that the Gompertz model, which is generally used for the interpretation of population growth (Tjørve and Tjørve, 2017), can well predict the longitudinal patterns of aerenchyma formation in rice roots.

To evaluate age-dependent aerenchyma formation in the nodal roots of *Z. nicaraguensis*, the Gompertz models were fitted to the percentage of aerenchyma formed at 10–80 mm from the root tips (**Figure 2C**). To convert the distance from the root tips to time after root-cell emergence (age), the distance from the root tips was calculated every 1 h based on the root elongation rate (control; 1.86 mm h^−1^, IAA treatment; 1.38 mm h^−1^, NPA treatment; 1.31 mm h^−1^, IAA and NPA treatment; 1.34 mm h^−1^) (**Figure 2A**). Subsequently, this explanatory variable was used in the Gompertz models to evaluate age-dependent aerenchyma formation (**Figure 2D**). We found that the IAA treatment negatively affected age-dependent aerenchyma formation in *Z. nicaraguensis*, similar to the effect on aerenchyma formation in rice roots (Yamauchi et al., 2019b). In contrast, the analyses of age-dependent aerenchyma formation confirmed the reduction in aerenchyma formation by NPA treatment and the restoration of this reduction by IAA treatment (**Figure 2D**).

### Analysis of the transverse pattern of lateral root distribution in the rotated nodal roots

**Figure 3:**
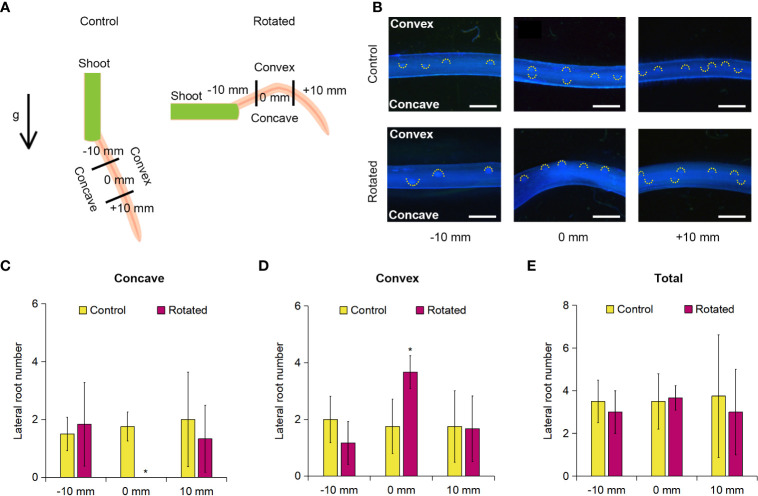
Lateral root formation on the concave and convex sides of the gravity-stimulated nodal roots of *Z. nicaraguensis*. **(A)** Diagram of gravistimulation experiment. The arrow indicates the direction of gravity (g) during the control and rotated treatments. **(B)** Pictures of lateral root primordia in the control and rotated nodal roots in the bent region (0 mm ± 2 mm) and at 10 mm (± 2 mm) from the bending position (at 0 mm). The basal and apical directions from the upright position are indicated with minus (−) and plus (+) signs, respectively. LR primordia were visualized using DAPI staining. Scale bars = 1 mm. Numbers of lateral root primordia at −10, 0, and +10 mm on the concave side **(C)** and convex side **(D)** and the total number of lateral root primordia in the nodal roots **(E)**. Significant differences between the control and rotated nodal roots at *p* < 0.05 (two-sample *t-*test) are denoted with asterisks. Values are mean ± SD (*n* = 3 to 4).

LR formation is correlated with CA formation in rice roots in relation to their auxin-dependent spatial distribution (Yamauchi et al., 2019b). Therefore, we first examined the transverse pattern of LR distribution in the rotated nodal roots of *Z*. *nicaraguensis*. For this analysis, 20-day-old aerobically grown *Z. nicaraguensis* seedlings, in which the roots were removed from the shoots (except for the single third nodal roots), were fixed on a 1.0% agar plate, and then the plate was placed perpendicular to the vertical plane for 72 h in the rotated treatment (**Figure 3A**). The control seedlings were kept parallel to the vertical plane for 72 h (**Figure 3A**). The root tips were marked as 0 mm when the treatment was initiated, and 10 mm from 0 mm toward the basal and apical parts were defined as −10 mm and +10 mm, respectively (**Figure 3A**).

Gravistimulation (rotation) treatments result in higher auxin levels in the pericycle on the convex side of the rotated roots immediately before the initiation of LR primordia in *Arabidopsis* and rice (Ottenschläger et al., 2003; Lucob-Agustin et al., 2020). We found that the number of LR primordia detected with DAPI fluorescence was higher on the convex side than on the concave side of the rotated nodal roots (**Figure 3B**). Therefore, we divided the nodal roots into concave and convex sides and then counted the LR primordia on each side of the nodal roots. There was a significant decrease in the number of LR primordia at 0 mm on the concave side of the rotated roots compared with that of the control roots (**Figure 3C**). In contrast, the number of LR primordia on the convex side of the rotated roots was significantly higher than that of the control roots (**Figure 3D**). Interestingly, there was no significant difference in the total number of LR primordia between the rotated and control roots at 0 mm (±2 mm; **Figure 3E**). During the 72-h treatment, both control and rotated roots elongated by approximately 60 mm, indicating that the bent part (0 mm) corresponds to the 60-mm part from the root tips. As the total number of LR primordia at 0 mm (±2 mm) was comparable with that in the control nodal roots at 60 mm from the root tips under aerated conditions (**Figure 3E** and **Supplementary Figure S3**), the removal of the roots by cutting did not affect LR formation in the remaining nodal roots. At −10 mm (±2 mm) and +10 mm (±2 mm), no significant differences in the number of LR primordia were detected between the rotated and control roots (**Figures 3C–E**).

### Analysis of the transverse pattern of aerenchyma formation in the rotated nodal roots

**Figure 4:**
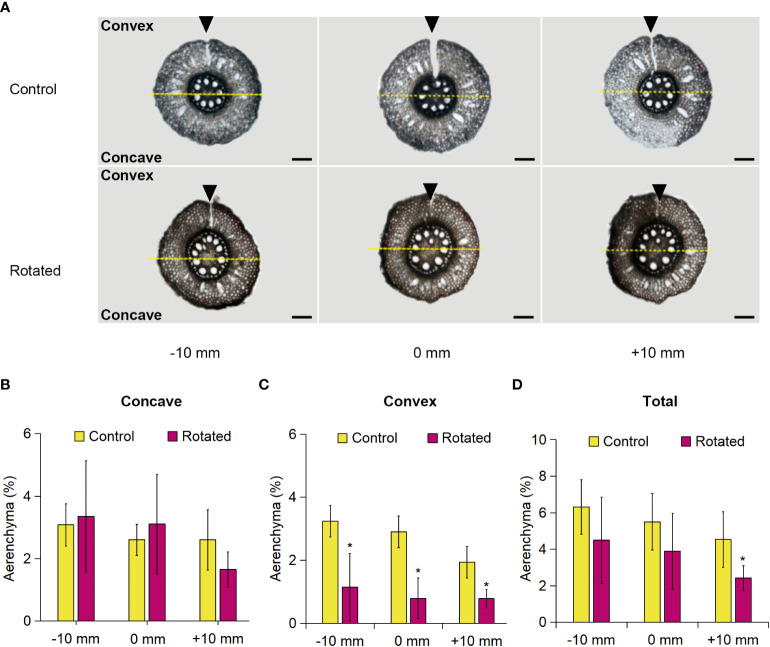
Aerenchyma formation on the concave and convex sides of the gravity-stimulated nodal roots of *Z. nicaraguensis*. **(A)** Cross-sections of the control and rotated nodal roots in the bent region (0 mm ± 2 mm) and at 10 mm (± 2 mm) from the bending position (at 0 mm). The parts cut by a razor blade are indicated with arrowheads. Scale bar = 1 mm. The percentage of aerenchyma formed at −10, 0, and +10 mm on the concave side **(B)** and convex side **(C)** and the total percentage of aerenchyma formed in the nodal roots **(D)**. Significant differences between the control and rotated nodal roots at *p* < 0.05 (two-sample *t-*test) are denoted with asterisks. Values are mean ± SD (*n* = 6).

The transverse pattern of aerenchyma formation in the roots during gravistimulation remains unclear. For clarification, we analyzed aerenchyma formation within the rotated third nodal roots of *Z. nicaraguensis*. To distinguish the concave and convex sides of the root cross-sections, we marked the top of the root on the convex side by cutting with a razor blade immediately before sampling and then separately analyzed the percentage of aerenchyma formed on the concave and convex sides (**Figure 4A**). The percentage of aerenchyma formed on the concave side of the rotated roots at −10, 0, and +10 mm was comparable to that of the control roots (**Figure 4B**), whereas the percentage on the convex side of the rotated roots was significantly decreased (**Figure 4C**). There was no significant difference between the total percentage of aerenchyma formed between the control and rotated roots, except for the percentage at +10 mm (**Figure 4D**). The total percentage of aerenchyma formed at 0 mm was comparable with that in the control nodal roots at 60 mm from the root tips under aerated conditions (**Figures 1B**, **4D**), supporting that the removal of the roots by cutting did not affect aerenchyma formation in the remaining nodal roots.

### Time-course analysis of the transverse pattern of aerenchyma formation in the rotated nodal roots

**Figure 5:**
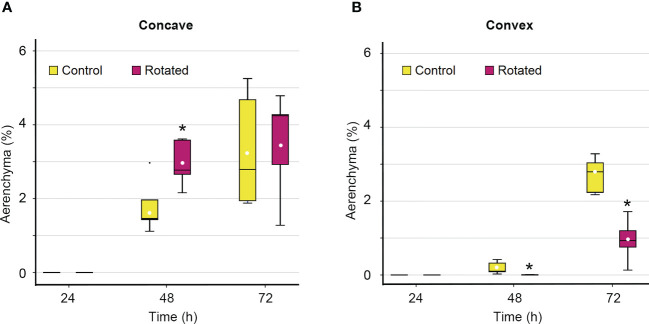
Time-course of aerenchyma formation in the cortex on the concave and convex sides in the bent region (0 mm ± 2 mm) of the gravity-stimulated nodal roots of *Z. nicaraguensis*. Percentage of aerenchyma formed on the concave side **(A)** and convex side **(B)** of the nodal roots at 24, 48, and 72 h after the initiation of the treatment. Boxplots show the median (horizontal lines), 25^th^ to 75^th^ percentiles (edge of the boxes), minimum to maximum (edge of the whiskers), and mean values (dots in the boxes) (*n* = 5). Significant differences between the control and rotated nodal roots at *p* < 0.05 (two-sample *t-*test) are denoted with asterisks.

Although the percentage of aerenchyma formed on the concave side of the rotated roots was not significantly different from that of the control roots 72 h after treatment initiation (**Figure 4B**), there is a possibility that the timing of aerenchyma formation is affected by gravistimulation. To test this hypothesis, a time-course analysis of the percentage of aerenchyma formed was performed on the concave and convex sides of the third nodal roots at 0 mm. While aerenchyma formation was first detected at 48 h on the concave side of the control and rotated roots, the percentage of aerenchyma formed was significantly higher in the rotated roots than that in the control roots (**Figure 5A**). A low percentage of aerenchyma formation on the convex side of the rotated roots was also detected at 48 h after treatment initiation (**Figure 5B**).

### Time-course expression analysis of auxin-responsive genes in the cortex of rotated nodal roots

**Figure 6:**
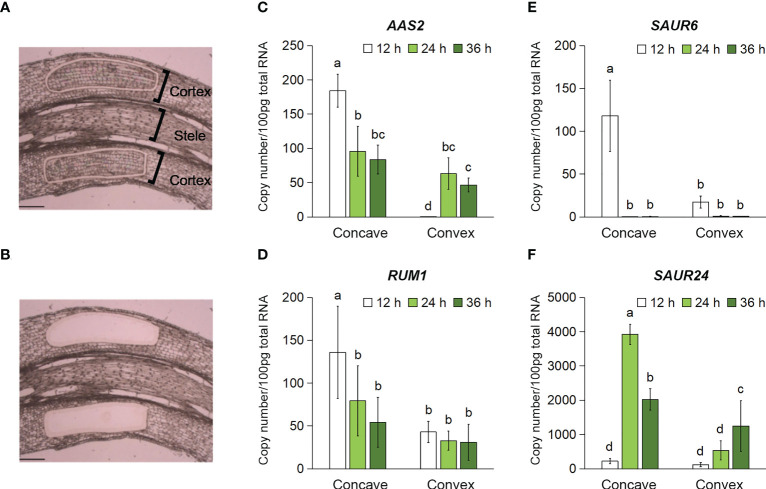
Time-course expression levels of the auxin-responsive genes in the cortex on the concave and convex sides in the bent region (0 mm ± 2 mm) of the gravity-stimulated nodal roots of *Z. nicaraguensis*. Pictures of the longitudinal section of the nodal roots before **(A)** and after **(B)** the isolation of the cortex using laser microdissection. Scale bar = 300 µm. Copy numbers of the transcripts of *AAS2* **(C)**, *RUM1* **(D)**, *SAUR6* **(E)**, and *SAUR24* **(F)** in the cortex on the concave and convex sides at 12, 24, and 36 h after the gravistimulation treatment. Significant differences between the concave and convex sides at each time point are denoted with different lowercase letters (*p* < 0.05, one-way ANOVA, followed by LSD test for multiple comparisons). Values are mean ± SD (*n* = 3 to 5).

A time-course analysis of aerenchyma formation revealed that it was stimulated and suppressed on the concave and convex sides of the rotated nodal roots 48 h after treatment initiation (**Figure 5**). To predict the auxin distribution pattern in the cortex of the rotated nodal roots, the cortex-specific expression of auxin-responsive genes was analyzed using tissues collected from the cortex on the concave and convex sides of the rotated nodal roots at 0 mm (±2 mm) using LM (**Figures 6A, B**). Among the auxin-responsive genes, we selected *ZmAAS2/GH3* (auxin amido synthetase 2; Zm00001d006753; Feng et al., 2015), *ZmRUM1* (rootless with undetectable meristems 1; Zm00001d043878; von Behrens et al., 2011), and *ZmSAUR6* (small auxin-up RNA family members; Zm00001d032094; Chen et al., 2014) and *ZmSAUR24* (Zm00001d006101; Chen et al., 2014), because their expression in maize roots is induced by auxin (von Behrens et al., 2011; Chen et al., 2014; Feng et al., 2015). We analyzed the transcript levels of the homologs of these auxin-responsive genes in *Z. nicaraguensis* at 12, 24, and 36 h after treatment initiation to clarify the timing of auxin signal activation (**Figures 6C–F**). The results showed that the transcript levels of *AAS2*, *RUM1*, *SAUR6*, and *SAUR24* were significantly higher on the concave side than on the convex side of the cortex (**Figures 6C–F**). Moreover, in the cortex on the concave side of the rotated roots, the transcript levels of *AAS2*, *RUM1*, and *SAUR6* peaked at 12 h (**Figures 6C–E**), and the transcript level of *SAUR24* peaked at 24 h (**Figure 6F**). Interestingly, in the cortex on the convex side, the expression of *AAS2* peaked at 24 h (**Figure 6C**), and that of *ZmSAUR24* gradually increased over time until 36 h (**Figure 6F**). These results were consistent with the fact that aerenchyma formation was not completely suppressed or inhibited on the convex side of the roots (**Figures 4C**, **5B**). The results suggest that asymmetric auxin distribution is essential for determining the contrasting pattern of aerenchyma formation in the rotated nodal roots of *Z*. *nicaraguensis*.

## Discussion

Our results demonstrate that auxin is involved in CA formation in the nodal roots of *Z. nicaraguensis* (**Figures 1**, **2**). Although the endogenous auxin IAA stimulated aerenchyma formation at the same distances from the root tips (**Figure 2C**), the opposite results were obtained through an age-dependent analysis (**Figure 2D**). In a previous study (Yamauchi et al., 2019b), CA formation in rice roots was reduced by exogenous IAA treatment, and this observation is consistent with the results obtained here from the age-dependent analysis of CA formation in *Z*. *nicaraguensis* roots (**Figure 2D**). This finding shows that an age-dependent evaluation is more effective in revealing accurate patterns of aerenchyma (Yamauchi and Nakazono, 2022b). The polar auxin transport inhibitor NPA suppressed aerenchyma formation, and co-treatment with IAA partially restored its formation (**Figure 2D**). These results suggest that a longitudinal auxin gradient is required for CA formation in the roots of wetland species (e.g., rice and *Z. nicaraguensis*). Dubrovsky et al. (2011) showed that LR primordia are initiated at the end of the elongation zone in the primary roots of *Arabidopsis*, where the auxin level is considerably lower than that in the most apical part. An auxin minimum and a subsequent increase in auxin level are also required for CA formation in rice roots (Yamauchi et al., 2019b). Consequently, owing to the cancellation of the auxin minimum, exogenous IAA suppresses CA formation, and the failure of increase in the auxin level in the presence of NPA is partially compensated by IAA (**Figure 2D**). The conservation of the mechanisms underlying CA formation in rice and *Z. nicaraguensis* suggests that auxin flux and signaling are feasible for plants to establish longitudinal patterns of CA formation, as is the case with LR formation (Dubrovsky et al., 2011; Xuan et al., 2016).

Auxin flow in the direction of gravity enables plant roots to achieve continuous downward growth (Ottenschläger et al., 2003). In the gravistimulation experiment, we identified an asymmetric pattern of LR formation in the bent region (at 0 ± 2 mm), that is, a decrease and increase in the number of LR primordia on the concave and convex sides of the nodal roots, respectively (**Figures 3C, D**). The total number of LR primordia was comparable between the concave and convex sides (**Figure 3E**), suggesting that an asymmetric auxin distribution underlies the contrasting pattern of LR formation. Similar observations have been reported in rice and *Arabidopsis*, where a transient increase in auxin levels in the pericycle triggers LR formation on the upper (convex) side of rotated roots (Richter et al., 2009; Lucob-Agustin et al., 2020). By contrast, the auxin levels in the cortex transiently increase on the lower (concave) side of the rotated roots of *Arabidopsis* (Ottenschläger et al., 2003). The results of this study showed that CA formation in the cortex was accelerated by gravistimulation (**Figure 5A**). This finding suggests that the transient increase in the auxin level in the cortex provides a local auxin source to stimulate CA formation.

The cortex-specific expression analysis using LM revealed that the auxin-responsive genes *AAS2*, *RUM1*, *SAUR6*, and *SAUR24* were highly expressed in the cortex on the concave side of the rotated nodal roots (**Figure 6**). Moreover, the expression of *AAS2*, *RUM1*, and *SAUR6* peaked at 12 h after the initiation of gravistimulation (**Figures 6C–E**). This finding is reasonable considering that the auxin level on the concave side of the rotated root of *Arabidopsis* transiently increases 5 h after gravistimulation (Ottenschläger et al., 2003). The expression of *AAS2* and *SAUR24* on the convex side of the rotated nodal roots peaked at 24 and 36 h, respectively (**Figures 6C, F**). This finding indicates a low level of auxin in the cortex on the convex side of the rotated nodal roots. Indeed, some extent of aerenchyma formation was detected in the cortex on the convex side (**Figure 4C**). It should be noted that auxin distribution in the cortex was successfully predicted using LM-isolated tissues, despite the limitations of generating transgenics of *Z. nicaraguensis*.

**Figure 7:**
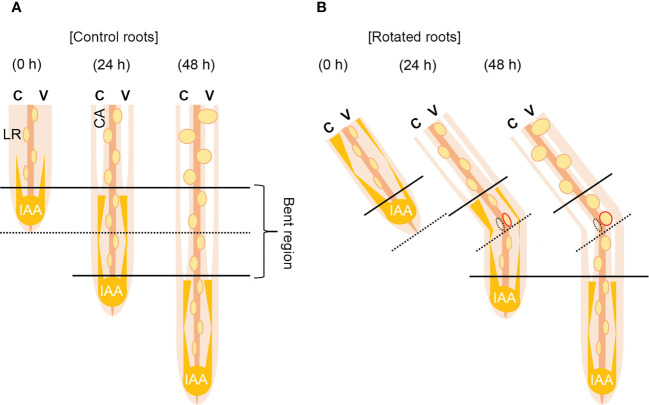
Model of contrasting aerenchyma formation regulated by asymmetric auxin distribution in the cortex of the gravity-stimulated nodal roots of *Z. nicaraguensis*. **(A)** Lateral root (LR) and constitutive aerenchyma (CA) formation is symmetric in the control. In the cortex, longitudinal gradients of auxin stimulate CA formation. **(B)** LR and CA formations are asymmetric under the gravistimulation treatment. Auxin levels increase and decrease in the cortex on the concave and convex sides, respectively. Thus, CA formation is accelerated on the concave side and retarded on the convex side of the nodal roots. By contrast, a transient increase in the auxin level on the convex side of the pericycle promotes LR formation. C; concave side, V; convex side.

Based on these results, we proposed a model for auxin-dependent CA formation in the rotated nodal roots of *Z. nicaraguensis* (**Figure 7**). A longitudinal auxin gradient is required for CA formation in the nodal roots (**Figures 7A, B**). After gravistimulation, the auxin level increases in the cortex on the concave side of the rotated nodal roots, and the elevated auxin level triggers CA formation (**Figure 7B**). By contrast, the decrease in auxin level in the cortex on the convex side results in a reduction in CA formation (**Figure 7B**). Simultaneously, the contrasting pattern of auxin distribution in the pericycle may reduce and stimulate LR formation on the concave and convex sides of the rotated nodal roots, respectively (**Figure 7B**). Overall, CA and LR formation in the bent region of the nodal roots of *Z. nicaraguensis* is coordinately regulated through gravity-induced auxin redistribution. However, we cannot exclude the possibility that LR formation provides a diffusion path to decrease the local ethylene level in the cortex on the convex side, thereby preventing aerenchyma formation as previously reported (Evans, 2003). Further studies are therefore required to understand the physiological mechanisms underlying the interaction between CA formation and LR formation in the main roots.

In plants, the auxin-dependent regulation of root gravitropism and LR initiation improves the utilization efficiency of soil resources. On the concave side of the bent roots, resources become depleted because of uptake by dense root hairs, whereas on the convex side with sparse root hairs, LRs more efficiently capture water and nutrients (Lucas et al., 2008). However, the benefit of coordinating the regulation of LR and CA formation is unknown. In the present study, we found that CA formation occurred on the opposite side of LR formation (**Figures 3C, D**, **5A, B**), suggesting that CA formation in the cortex on the concave side of the nodal roots contributes to a reduction in respiratory costs and the allocation of resources for the outward LR growth. In fact, aerenchyma formation has been demonstrated to reduce the respiratory cost of the roots (Lynch, 2018; Lynch, 2019; Schneider et al., 2020a; Schneider et al., 2020b). In addition, increased aerenchyma formation in the recombinant inbred lines of maize has been found to correlate with increased root length density in deep soil layers under drought conditions (Zhu et al., 2010). Two simulation studies have predicted that aerenchyma contributes to altered carbon allocation within the plant resulting in longer root lengths and is beneficial for nutrient acquisition, particularly under nutrient deficiency (Postma and Lynch, 2011a; Postma and Lynch, 2011b). A similar mechanism may underlie aerenchyma formation during gravistimulation. By contrast, the reduction in CA formation on the convex side of the rotated roots may contribute to maintaining the mechanical strength of the roots that are negatively affected by the penetration of LRs.

Another line of evidence supporting the correlation between aerenchyma and LR formation is the hydropatterning response, in which the main roots perceive the spatial distribution of water availability in soils (Yu et al., 2016; Orosa-Puente et al., 2018). Hydropatterning is established by increasing auxin levels on the waterside, which triggers LR initiation (Bao et al., 2014; Robbins and Dinneny, 2018). Interestingly, aerenchyma formation has been detected on the opposite (dry) side of the main roots of rice and maize (Bao et al., 2014; Yu et al., 2016; Robbins and Dinneny, 2018). Although the function of aerenchyma formation during hydropatterning remains unclear, balancing of resource allocation by aerenchyma formation may contribute to water acquisition by newly formed LRs. It should be noted that aerenchyma formation has been detected on the dry side of maize roots (Robbins and Dinneny, 2018), which hardly forms CA, suggesting that this aerenchyma formation is induced by auxin. Justin and Armstrong (1991) reported that auxin stimulates ethylene production and induces aerenchyma formation in the roots of maize. Moreover, auxin can stimulate ethylene biosynthesis and inducible aerenchyma formation in rice roots (Yamauchi et al., 2020b). These observations suggest that at least some part of the aerenchyma formation process during gravistimulation and the hydropatterning response is auxin-dependent but ethylene-mediated (inducible), and it resembles root cortical senescence in barley, which is also modulated by ethylene (Schneider et al., 2018). Further studies are therefore required to understand the detailed mechanisms underlying flooding-independent aerenchyma formation.

## Conclusion

Our study revealed that CA formation in the nodal roots of *Z*. *nicaraguensis* is regulated by auxin. Moreover, CA formation during gravistimulation is coordinately regulated with LR formation through tissue-specific auxin redistribution in the nodal roots of *Z*. *nicaraguensis*. This mechanism could contribute to the normal root growth of plants and their responses to abiotic stresses. CA formation in the cortex on the concave side of the rotated roots may balance resource allocation and thereby contribute to water and nutrient uptakes by outward-grown LRs. This concept will promote a deeper understanding of auxin-dependent asymmetric root development during gravistimulation and the hydropatterning response. As *Z*. *nicaraguensis* can be crossed with maize to form constitutive aerenchyma, it is a reliable genetic resource to optimize the root architecture of maize and thus improve productivity.

## Data availability statement

The original contributions presented in the study are included in the article/**Supplementary Material**. Further inquiries can be directed to the corresponding author.

## Author contributions

JN performed the experiments, analyzed the data, and wrote the draft of the manuscript. JN, HT, and MN designed the experiments. TY, HT, and MN supervised the experiments. TY, HT, FO, YM, and MN revised the manuscript. All authors contributed to the article and approved the submitted version.
