# Supplementary material for: Asymmetric auxin distribution establishes a contrasting pattern of aerenchyma formation in the nodal roots of Zea nicaraguensis during gravistimulation
Source: Front Plant Sci. 2023 Apr 19;14:1133009. doi: 10.3389/fpls.2023.1133009 (PMC10154625; doi:10.3389/fpls.2023.1133009)
Supplement: Supplementary file 1 [file DataSheet_1.pdf]

Supplementary Material

Table S1 | List of primers for qRT-PCR analysis

	Forward primer	Reverse primer
AAS2	CCAGTTAGAAGCGTGAGGGTAA	AAGCAGAAGCAGCCCAACTC
RUM1	GAATCGTCAAAACCCGTCAAG	CGGATCACGTCACAGACATACA
SAUR6	TCCTTCCTCCCCTGTTCTTGT	CCTCTCTCTCTCTCCCTCCTTC
SAUR24	ATGGCCACGGAGAAAGGAAG	CCCAGCGTCTTCGTGATGAG

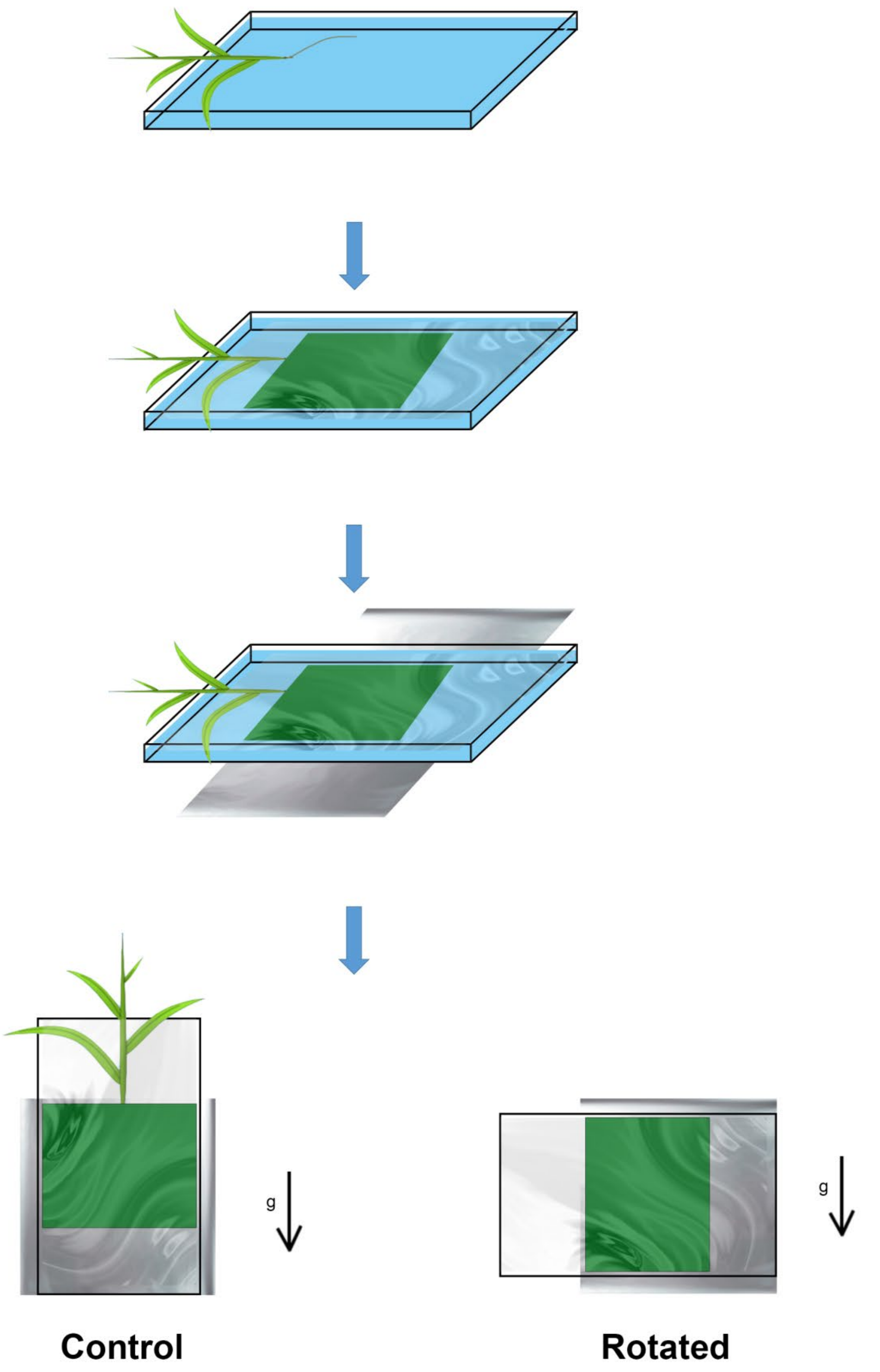

2

Figure S1 | Scheme of the gravistimulation treatment using agar plates. The single third nodal roots (of lengths 30–40 mm) were selected from 20-day-old *Z. nicaraguensis* seedlings, and the remaining roots were removed by cutting with a pair of forceps. The seedlings were fixed on uncovered 1.5-L plastic transparent plates containing 1.0% (w/v) agar. The root parts were covered with a wet paper towel and plastic wrap to prevent drying and then covered with aluminum foil. The nodal roots were kept perpendicular to the vertical plane for the gravistimulation treatment and parallel to the vertical plane for the control treatment.

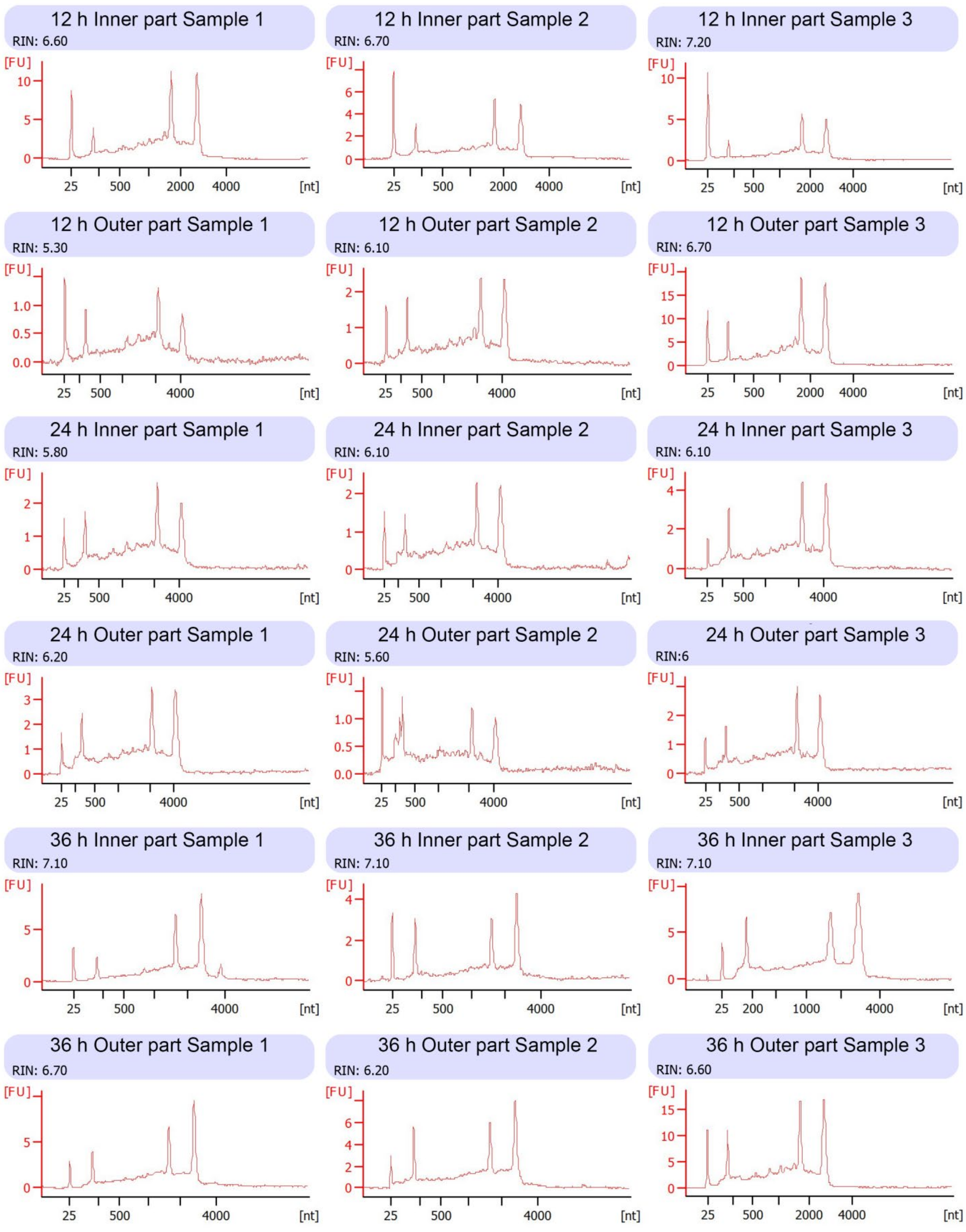

Figure S2 | Electropherogram images of total RNA extracted from LM-isolated tissues. RNA quality is expressed as RNA integrity number (RIN); RNA quality was determined using an Agilent 2100

Bioanalyzer. The X-axis represents the size of fragments (nt: nucleotide) and the Y-axis represents the wavelength of fluorescence (FU: fluorescence unit).

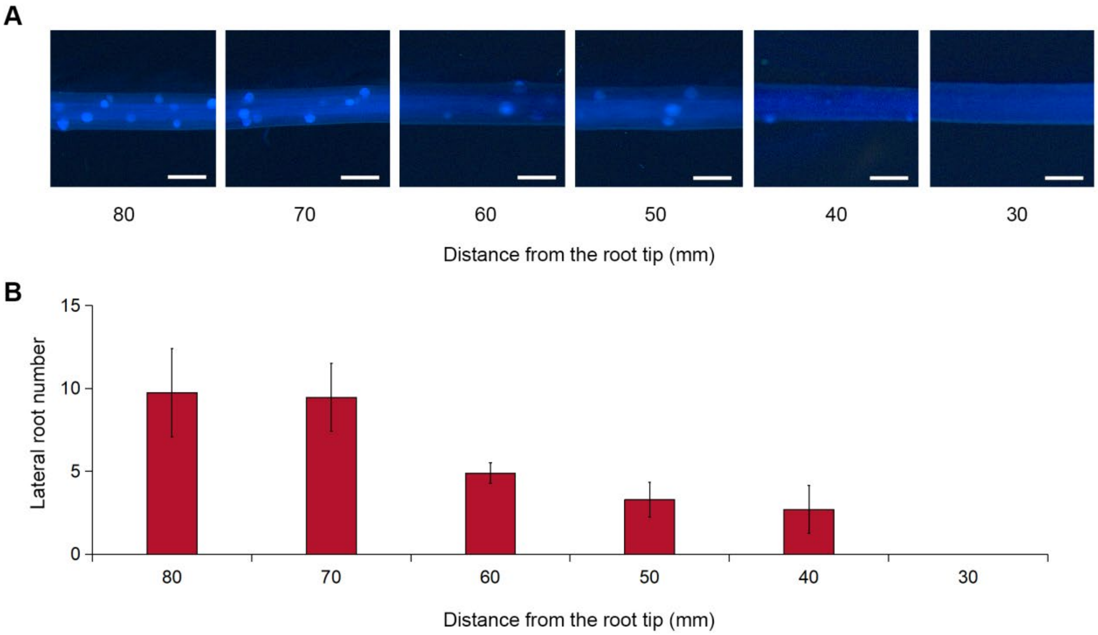

Figure S3 | Lateral root formation in the third nodal roots of *Z. nicaraguensis* under aerated conditions. **(A)** Pictures of DAPI-stained lateral root primordia at 30, 40, 50, 60, 70, and 80 mm (± 2 mm) from the tips of third nodal roots. Scale bar = 1 mm. **(B)** Number of lateral root primordia at 30, 40, 50, 60, 70, and 80 mm (± 2 mm) from the tips of the third nodal roots. Values are mean ± SD (n = 3 to 4).
